# Bioinformatic analysis of the effect of SNPs in the pig *TERT* gene on the structural and functional characteristics of the enzyme to develop new genetic markers of productivity traits

**DOI:** 10.1186/s12864-023-09592-y

**Published:** 2023-08-25

**Authors:** Mykyta Peka, Viktor Balatsky, Artem Saienko, Oleksandr Tsereniuk

**Affiliations:** 1 Institute of Pig Breeding and Agroindustrial Production, National Academy of Agrarian Sciences of Ukraine, 1 Shvedska Mohyla St, Poltava, 36013 Ukraine; 2 V. N. Karazin Kharkiv National University, 4 Svobody Sq, Kharkiv, 61022 Ukraine

**Keywords:** Telomerase reverse transcriptase, Bioinformatic analysis, Prediction, Genetic markers, Folding free energy

## Abstract

**Background:**

Telomerase reverse transcriptase (TERT) plays a crucial role in synthesizing telomeric repeats that safeguard chromosomes from damage and fusion, thereby maintaining genome stability. Mutations in the *TERT* gene can lead to a deviation in gene expression, impaired enzyme activity, and, as a result, abnormal telomere shortening. Genetic markers of productivity traits in livestock can be developed based on the *TERT* gene polymorphism for use in marker-associated selection (MAS). In this study, a bioinformatic-based approach is proposed to evaluate the effect of missense single-nucleotide polymorphisms (SNPs) in the pig *TERT* gene on enzyme function and structure, with the prospect of developing genetic markers.

**Results:**

A comparative analysis of the coding and amino acid sequences of the pig *TERT* was performed with corresponding sequences of other species. The distribution of polymorphisms in the pig *TERT* gene, with respect to the enzyme’s structural-functional domains, was established. A three-dimensional model of the pig TERT structure was obtained through homological modeling. The potential impact of each of the 23 missense SNPs in the pig *TERT* gene on telomerase function and stability was assessed using predictive bioinformatic tools utilizing data on the amino acid sequence and structure of pig TERT.

**Conclusions:**

According to bioinformatic analysis of 23 missense SNPs of the pig *TERT* gene, a predictive effect of rs789641834 (TEN domain), rs706045634 (TEN domain), rs325294961 (TRBD domain) and rs705602819 (RTD domain) on the structural and functional parameters of the enzyme was established. These SNPs hold the potential to serve as genetic markers of productivity traits. Therefore, the possibility of their application in MAS should be further evaluated in associative analysis studies.

## Background

Telomeres are short tandem nucleotide repeats with the TTAGGG motif in vertebrates [1, 2] located at the ends of chromosomes. The role of telomeres is to protect chromosomes from the destructive action of DNases and prevent their fusion, which is critical for maintaining the stability of the genome. The synthesis of telomeric repeats is carried out by telomerase, which is a ribonucleoprotein complex consisting of telomerase reverse transcriptase (TERT) and telomerase RNA (TER). TER acts as a template for the synthesis of telomeric tandem repeats which is carried out by TERT. The telomerase complex includes a number of other components that interact with the enzyme and are necessary for its functioning in cells [3]. The efficiency of telomerase is determined by the number of telomeric DNA repeats that it can complete at the ends of telomeres [4]. TERT enzyme is encoded by the corresponding *TERT* gene which is represented by orthologues in a wide range of biological species accordingly to the Ensembl database [5].

A decrease in the length of telomeres in mammalian somatic cells is associated with their division and is a consequence of end-replication problem, as well as blocking or decreasing telomerase activity. In contrast, in generative and stem cells, the enzyme activity remains high, which allows for maintaining the size of telomeric regions in a number of cell generations [2, 6]. However, under the influence of adverse environmental factors, a reduction in the number of telomeric repeats is often observed, and the length of telomeres in such cases can be considered a molecular indicator of such impact [7–11]. On the other hand, the shortening of the telomere length can obviously be caused by abnormal insufficient telomerase activity, which either initially does not allow the synthesis of the number of telomeric repeats typical for different cells of the organism or their number is not maintained in the generations of proliferating cells. One of the reasons for the latter may be mutations in the *TERT* and *TER* genes, some of which can cause both structural and functional changes in the telomerase complex and deviations in the level of expression of these genes [12–15].

Interest in studying the organization of telomeres, the structure of telomerase and the activity of this enzyme in cells of different tissues of the organism is associated, first, with the solution of fundamental problems of biology, the study of the mechanisms of cell division and apoptosis, the determination of the life span of the organism, and in the applied aspect, with the study of the causes and processes of the appearance cancerous tumors and a number of other diseases, the search for antitumor agents, the treatment of pathologies caused by abnormalities in the structure of telomeric regions, as well as the assessment of the “genetic age” of cloned animals [16].

In recent years, several studies have emerged that explore the relationship between telomere size, telomerase activity, and key qualities of livestock. These studies have considered such crucial parameters as animal health, productive lifespan, and resistance to stress in chickens [11], cattle [9, 17, 18], sheep [19], pigs [20], horses and donkeys [21]. Breeding progress in improving these qualities using traditional methods is limited by their low level of heritability and difficulty assessing them. At the same time, one of the approaches to accelerate such progress may be the use of molecular markers, which can be the length of telomeric repeats (the coefficient of their heritability for different species is determined in the range of 0.3 – 0.8) [17, 22–24] and genetically determined telomerase activity. In the context of the above, the polymorphisms found in *TERT* could be the basis for the development of genetic markers capable of showing association with key traits of livestock. This thesis is confirmed by studies where *TERT* is considered a candidate gene related to a number of physiological and pathological processes [25–28] and identified in QTLs [29, 30].

Most often, the nature of genetic markers and candidate genes is associated with linkage disequilibrium with causative mutations that are the direct reason of phenotypic changes. The search for such causative mutations is associated with the transition to the study of individual polymorphisms and is a rather difficult task [31–33]. According to our suggestion, bioinformatic analysis methods can become a tool that facilitates its solution, which provides a preliminary screening of missense mutations and the selection of those that can potentially be causative. A similar bioinformatics-based approach has previously been successfully used to identify candidate disease genes and predict the potential impact of polymorphic variants on human phenotype [34, 35], and therefore can be applied to the analysis of genetic polymorphisms in animals. The use of this bioinformatic approach can precede experimental testing of polymorphisms, thereby reducing the amount of laboratory work required.

Considering pig *TERT* in our study as an object for the development of genetic markers, one should pay attention to the fact that many SNPs were found in the gene of this species (674 allelic variants of the gene were known for pig *TERT* according to the Ensembl database) some of them are the result of missense substitutions. Bioinformatic tools can be used for predictive assessment of their influence and for narrowing the range of SNPs that claim to be genetic markers. Bioinformatic analysis makes it possible to calculate the effects of missense variants on the enzyme structure and function. This will help determine the most promising polymorphisms, which can be further considered probable genetic markers and for which it is advisable to conduct association studies for MAS.

This study proposes an analysis of the structure of the pig *TERT* gene in comparison with other biological species, as well as a prognostic evaluation of the influence of missense SNPs found in the pig *TERT* gene on the structural and functional characteristics of the enzyme with the prospect of developing genetic markers for MAS of this animal species.

## Results

### Comparative analysis of the CDS and AAS of pig *TERT* with other biological species

*TERT* gene in pigs is mapped on chromosome 16 and includes 17 exons with a transcript size of 5497 bp. The length of pig *TERT* AAS is 1130 amino acid residues accordingly to the reference sequence from the Ensembl database [5]. In order to gain a more complete idea about the pig *TERT* gene, a comparative analysis was carried out with the human *TERT* gene, which is rather well studied, and corresponding orthologues of some animals that are important model or livestock species. The results of the pairwise alignment of the pig *TERT* CDS and AAS with the corresponding sequences of other biological species indicate the highest degrees of identity and similarity with those species that are most phylogenetically close: cattle, sheep, goat, horse, donkey (Table 1). High degrees of identity and similarity were also found for the pig *TERT* CDS and AAS with human *TERT*. The smallest degrees of identity and similarity of pig *TERT* were observed with the *TERT* of those biological species that are phylogenetically distant from the pig, such as rodents (mouse, rat), and with the *TERT* of other common model organisms (African clawed frog, zebrafish, takifugu).

**Table 1:** Comparison of pig *TERT* CDS тa AAS with other biological species

Organisms	Protein length, aa	CDS identity, %	AAS identity, %	AAS similarity, %
Pig (*Sus scrofa*)	1130	—	—	—
Human (*Homo sapiens*)	1132	77.7%	71.8%	80.1%
**Model organisms:**				
Mouse (*Mus musculus*)	1122	66.1%	59.2%	71.5%
Rat (*Rattus norvegicus*)	1125	67.7%	59.2%	71.5%
African clawed frog (*Xenopus laevis*)	1191	45.6%	40.0%	57.0%
Zebrafish (*Danio rerio*)	1098	49.3%	33.6%	48.9%
Takifugu (*Takifugu rubripes*)	1074	46.2%	34.2%	49.6%
**Livestock:**				
Cattle (*Bos taurus*)	1125	81.0%	76.2%	82.9%
Sheep (*Ovis aries*)	1123	81.5%	75.9%	82.5%
Goat (*Capra hircus*)	1123	81.2%	75.5%	82.3%
Horse (*Equus caballus*)	1151	80.7%	75.9%	82.6%
Donkey (*Equus asinus*)	1146	81.2%	76.1%	82.8%

### Distribution of the polymorphisms in the pig *TERT* gene

Information regarding the number and chromosomal localization of the polymorphisms in the pig *TERT* gene was obtained from the Ensembl database in which known genetic variants are connected with corresponding rsIDs. The distribution of these polymorphisms was then established in relation to the gene regions responsible for encoding distinct structural-functional domains of the TERT enzyme (Table 2). For this analysis, a comparison was made between the pig *TERT* gene and its orthologues in cattle and humans. The selection of cattle as a comparative species was based on their phylogenetic proximity to pigs, while the choice of humans was because they are the most extensively studied species, and information on the domain structure of human TERT is available in the UniProt database [36]. This approach was implemented through multiple alignment of pig, human and cattle *TERT* AASs. Consequently, the expected localization of the four main domains (TEN, TRBD, RTD, CTE) and linker regions [6] in the pig telomerase reverse transcriptase molecule were determined. Subsequently, the pig polymorphisms were categorized into the respective regions of the pig TERT enzyme.

**Table 2:** Polymorphism of orthologues *TERT* genes

Human				Pig				Cow			
Domain/site	Number of PMs	% of total PMs in transcript	Density of PMs per bp	Domain/site	Number of PMs	% of total PMs in transcript	Density of PMs per bp	Domain/site	Number of PMs	% of total PMs in transcript	Density of PMs per bp
TEN (1–230)	336/178	20.60/19.0	0.487/0.258	TEN (1–228)	6/4	11.11/17.39	0.009/0.006	TEN (1–234)	75/56	13.89/14.74	0.107/0.080
Linker 1 (231–324)	191/131	11.71/13.98	0.677/0.465	Linker 1(229–319)	6/4	11.11/17.39	0.022/0.015	Linker 1(235–312)	27/16	5.00/4.21	0.115/0.068
TRBD (325–550)	330/199	20.23/21.24	0.487/0.294	TRBD(320–548)	12/5	22.22/21.74	0.017/0.007	TRBD(313–543)	63/44	11.67/11.58	0.091/0.063
Linker 2 (551–604)	57/31	3.49/3.31	0.352/0.191	Linker 2 (549–602)	1/0	1.85/0	0.006/0.0	Linker 2 (544–597)	20/15	3.70/3.95	0.123/0.093
RTD (605–935)	471/264	28.88/28.18	0.474/0.266	RTD (603–933)	19/5	35.19/21.74	0.019/0.005	RTD (598–928)	257/182	47.59/47.89	0.259/0.183
CTE (936–1132)	246/134	15.08/14.30	0.414/0.226	CTE (934–1130)	10/5	18.52/21.74	0.017/0.008	CTE (929–1125)	98/67	18.15/17.63	0.165/0.113
Total	1631/937	100.0	0.480/0.276	Total	54/23	100.0	0.016/0.007	Total	540/380	100.0	0.160/0.112

The first number in each cell is an indicator determined for all polymorphisms in a given area; the second number is an indicator only for missense polymorphisms

**Fig. 1:**
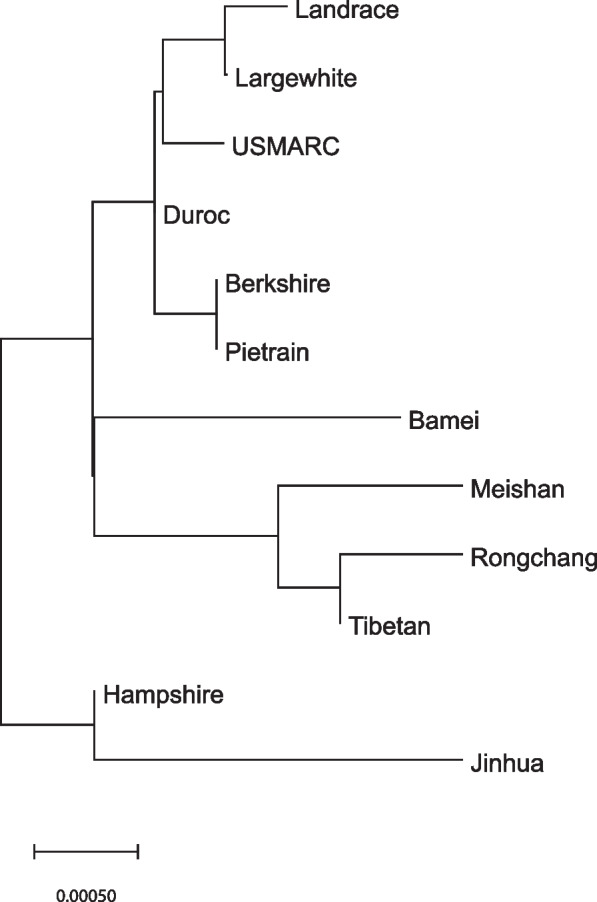
Phylogenetic tree of 12 pig breeds built based on the alignment of *TERT* coding sequences. Phylogenetic tree was built using the Maximum Likelihood method and the JTT matrix-based model based on the results of multiple alignment of *TERT* coding sequences performed according to the MUSCLE algorithm

Each of the regions of the *TERT* gene, corresponding to distinct structural-functional domains of telomerase reverse transcriptase, is characterized by a certain level of polymorphism. Both synonymous and missense SNPs occur. In order to assess the intraspecific variability of pig *TERT*, a phylogenetic analysis was carried out based on the CDSs of the *TERT* gene of 12 pig breeds, for which data on the whole genome sequencing are available. The phylogenetic tree presented in Fig. 1 reflects the results of this analysis.

It can be expected that the polymorphisms found in pig *TERT* can also affect the structure and telomerase activity of the enzyme. In prospect, polymorphisms with certain impact can be used to develop genetic markers for the biological and productive traits of pigs. In this study, only missense variants (23 pig SNPs with corresponding rsIDs from the Ensembl database) are considered because they change AAS and are able to introduce certain structural changes into the enzyme, so the potential impact of missense variants on TERT functional characteristics can be assessed using bioinformatic analysis methods. Data on pig *TERT* missense SNPs, their rsIDs, localization on the chromosome, corresponding allelic and amino acid variants are shown in Table 3. In addition, it is indicated which allelic variants according to these missense polymorphisms correspond to each of the analyzed 12 pig breeds.

**Table 3:** Missense SNPs in pig *TERT* gene

Domain	Variant ID (SNP)	Location on chromosome	Alleles	aa substitution	Pig breeds
TEN	rs789641834	16:79,259,174 Exon 2	C/A	L158M	C: **
	rs698799571	16:79,259,178 Exon 2	A/T	Y159F	A: **
	rs706045634	16:79,259,304 Exon 2	G/C	R201P	G: * C: meishan, rongchang, tibetan
	rs696805316	16:79,259,382 Exon 2	G/A	G227E	G: * A: berkshire, pietrain
Linker 1	rs338419951	16:79,259,489 Exon 2	G/A	D263N	G: * A: meishan
	rs318799866	16:79,259,510 Exon 2	A/G	T270A	A: * G: rongchang, tibetan
	rs705219838	16:79,259,511 Exon 2	C/T	T270I	C: **
	rs330770291	16:79,259,543 Exon 2	C/T	R281W	C: **
TRBD	rs325294961	16:79,259,762 Exon 2	C/G	R354G	C: **
	rs333763227	16:79,259,925 Exon 2	G/A	R408Q	G: **
	rs789588487	16:79,259,954 Exon 2	G/A	G418S	G: * A: rongchang
	rs324158660	16:79,260,249 Exon 2	T/C	I516T	T: * C: USMARC, landrace
	rs329843407	16:79,263,036 Exon 3	C/G	D523E	C: * G: rongchang
Linker 2	—	—	—	—	—
RTD	rs698738374	16:79,264,290 Exon 4	G/T	R606L	G: **
	rs705602819	16:79,264,358 Exon 4	C/T	R629W	C: **
	rs332450148	16:79,264,388 Exon 4	C/G	R639G	C: **
	rs339601952	16:79,265,184 Exon 5	G/A	A668T	G: **
	rs332245175	16:79,265,188 Exon 5	G/A	R669Q	G: **
CTE	rs787080565	16:79,272,561 Exon 14	G/A	A997T	G: **
	rs791792095	16:79,272,944 Exon 15	C/T	P1022L	C: **
	rs335037856	16:79,273,825 Exon 16	G/A	R1095Q	G: * A: hampshire, jinhua
	rs336411058	16:79,274,280 Exon 17	G/A	E1114K	G: * A: jinhua
	rs320614499	16:79,274,307 Exon 17	A/G	T1123A	A: * G: jinhua

^**^Corresponds to all studied pig breeds (bamei, berkshire, duroc, hampshire, jinhua, landrace, largewhite, meishan, pietrain, rongchang, tibetan, USMARC)

^*^Corresponds to all breeds, except for those specified by an alternative allele

Dashes (“—”) in the table indicate the absence of data about missense polymorphisms in Linker 2 entered into the Ensembl database. In the following tables, a separate line for Linker 2 was not used

### Prognostic evaluation of the effect of pig *TERT* gene missense SNPs on telomerase function and stability

The results of evaluating the impact of missense SNPs in the pig *TERT* gene on the function of the enzyme, obtained with sequence-based methods, are shown in Table 4. Combining a set of sequence-based predictive tools with various evaluation algorithms and synthesizing their results makes it possible to identify SNPs that are highly likely to have an effect on TERT enzyme and may be related to the productive traits of animals. Table 4 shows the numerical values ​​calculated by each of the predictive tools; if a certain threshold value is reached, they are considered to “have an effect” or “be deleterious (damaging)”, depending on the tool developer [37–44]. At the same time, it should be taken into account that the missense SNP with a pronounced effect on a protein molecule can be associated with both positive and negative functional consequences. Therefore, this study considers the division of SNPs by the results of sequence-based prediction into those that have a functional “effect” (or possible functional “effect”) and those that are “neutral” instead of characterizing “effective” SNPs as deleterious (damaging) ones.

**Table 4:** Prediction of the “effects” of missense SNPs on pig TERT protein

Domain	Variant ID (SNP)	aa substitution	SIFT	PROVEAN	PolyPhen-2	Panther	SNAP2
TEN	rs789641834	L158M	0.00**	–1.026	1.0**	0.5 (324 my)*	–1
	rs698799571	Y159F	1.0	0.575	0.018	0.17 (85bmy)	–45
	rs706045634	R201P	0.04**	–2.350	0.897*	0.19 (97 my)	67**
	rs696805316	G227E	0.2	–0.357	0.031	0.02 (1my)	–39
Linker 1	rs338419951	D263N	0.41	–0.618	0.693*	0.02 (1 my)	–27
	rs318799866	T270A	0.14	–0.716	0.522*	0.02 (2 my)	–24
	rs705219838	T270I	0.01**	–0.971	0.974**	0.02 (2 my)	10**
	rs330770291	R281W	0.09	0.216	0.004	0.02 (2 my)	47**
TRBD	rs325294961	R354G	0.00**	–3.914**	1.0**	—(not scored)^a^	53**
	rs333763227	R408Q	0.19	–1.555	0.99**	0.27 (176 my)	–45
	rs789588487	G418S	0.87	–0.221	0.006	0.02 (1 my)	–81
	rs324158660	I516T	0.47	0.956	0.002	0.02 (1 my)	–91
	rs329843407	D523E	0.11	-0.049	0.002	0.02 (1 my)	–41
RTD	rs698738374	R606L	0.19	-2.025	0.136	0.02 (1 my)	–2
	rs705602819	R629W	0.00**	-7.055**	1.0**	0.89 (1628 my)**	82**
	rs332450148	R639G	1.0	2.569	0.000	0.02 (1 my)	–43
	rs339601952	A668T	1.0	0.688	0.002	0.19 (97 my)	–96
	rs332245175	R669Q	0.52	–1.125	0.453*	0.19 (97 my)	–32
CTE	rs787080565	A997T	0.31	0.305	0.003	0.02 (1 my)	–89
	rs791792095	P1022L	0.08	–1.792	0.561*	0.19 (97 my)	4**
	rs335037856	R1095Q	0.25	–0.987	0.082	0.27 (176 my)	–27
	rs336411058	E1114K	1.0	–0.322	0.017	0.19 (97 my)	–75
	rs320614499	T1123A	0.94	–0.076	0.001	0.02 (1 my)	–80

The evaluation of the functional impact of the substitution is presented according to the criteria of each predictive tool: 1) SIFT: score < 0,05 — “deleterious”; score ≥ 0,05 — “tolerated”; 2) PROVEAN: score ≤ –2,5 – “deleterious”, score > –2,5 — “neutral”; 3) PolyPhen-2: score from 0.0 to 1.0 is a probability that substitution is “damaging” (“benign”, “possibly damaging” or “probably damaging”); 4) PANTHER: time < 200my — “probably benign”; 450my > time > 200my, — “possibly damaging” time > 450my — “probably damaging”; 5) SNAP2: score ≤ 0 — “neutral”, score > 0 — “effect”

^a^The indicator is not calculated by the program

^*^Indicates possibly damaging substitutions

^**^Indicates deleterious/damaging/effect substitutions

Tolerated/benign/neutral substitutions are not highlighted

Those SNPs for which the presence of an “effect” on TERT protein was predicted by the absolute majority of the tools include rs325294961 (R354G), and rs705602819 (R629W); among the SNPs for which such an “effect” is predicted by the vast majority of tools (or the value of the assessment is close to the threshold), rs789641834 (L158M) rs706045634 (R201P), and rs705219838 (T270I) should be considered.

**Fig. 2:**
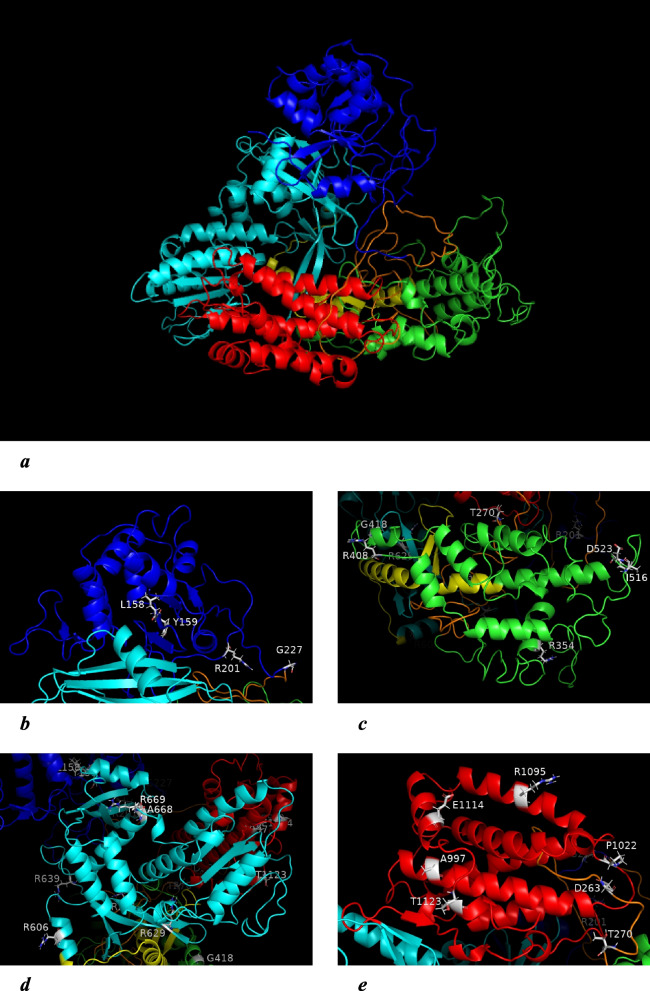
The tertiary structure of pig TERT (homology modeling). **a** – general view; **b** – TEN, **c** – TRBD, **d** – RTD, **e** – CTE. TEN domain is marked in blue, TRBD in green, RTD in cyan, CTE in red; Linker1 and Linker2 are marked in orange and yellow, respectively. Polymorphic amino acids are highlighted

Another approach to assess the effect of missense SNPs on the functional characteristics of TERT is structural analysis. For this purpose, the three-dimensional structure of the pig TERT was obtained by homology modeling on the basis of the corresponding structure of human TERT, which was made possible by a sufficient degree of identity between human and pig AASs (Table 1). Minimized variants of this structure were deposited and are free available (ModelArchive, accession codes: ma-ydbtw, ma-p89hn). The visualization of pig TERT structure is presented in Fig. 2. The localizations of amino acid substitutions corresponding to the considered missense SNPs in the *TERT* gene are also presented. It allows drawing a conclusion about the possible effect of each of the substitutions on the secondary and tertiary structures of a particular domain of the TERT protein.

The stability of the tertiary structure of the protein is ensured by its folding mechanism [45]. An important characteristic of protein stability is the level of its folding free energy [46]. In this study, the concept of folding free energy (ΔG) considers the difference between the Gibbs free energy between the unfolded (G_u_) and the folded (G_f_) states (ΔG = G_u_–G_f_); more positive values ​​of ΔG indicate greater energy stability of the protein [47]. To determine exactly how each of the studied missense SNPs affects the energy stability of the TERT protein, prediction of changes in folding free energy (ΔΔG) was made as the difference between the folding energy of protein with missense variant (ΔG_SNP_) and reference one (ΔG): ΔΔG = ΔG_SNP_ – ΔG_ref_. (Table 5). Substitutions that are characterized by negative ΔΔG values, thus, lead to a decrease in TERT protein stability, and substitutions for which the calculated value of folding free energy is positive are stabilizing.

**Table 5:** Changes in folding free energy (ΔΔG) under the effect of amino acid substitutions (kcal/mol)

Domain	Variant ID (SNP)	aa substitution	I-Mutant 3.0	PoPMuSiC	mCSM	SDM2	DDGun
TEN	rs789641834	L158M	–1.09	–0.94	–0.921	–1.1	–0.1
	rs698799571	Y159F	–0.96	–0.91	–1.053	–0.16	0.2
	rs706045634	R201P	–0.31	0.15	0.366	0.67	–0.0
	rs696805316	G227E	–0.45	0.71	–0.877	–0.72	0.0
Linker 1	rs338419951	D263N	–1.13	–0.33	–0.188	0.15	0.0
	rs318799866	T270A	–1.65	–1.37	–1.356	–0.48	–0.0
	rs705219838	T270I	–0.74	–0.56	–0.43	0.59	0.2
	rs330770291	R281W	0.01	–0.29	–0.423	0.24	0.1
TRBD	rs325294961	R354G	–1.7	–2.9	–1.585	–1.23	–0.3
	rs333763227	R408Q	–0.55	–0.09	0.187	–0.05	–0.0
	rs789588487	G418S	–0.77	0.03	–0.93	–0.2	0.1
	rs324158660	I516T	–1.18	–0.49	–0.741	–0.22	–0.1
	rs329843407	D523E	–0.34	–0.21	–0.851	0.07	0.0
RTD	rs698738374	R606L	0.04	0.29	0.299	0.3	0.0
	rs705602819	R629W	–0.03	–0.32	0.148	0.57	–0.0
	rs332450148	R639G	–0.32	–0.01	–0.083	0.16	–0.1
	rs339601952	A668T	–0.9	–0.32	–0.99	–1.53	0.1
	rs332245175	R669Q	–1.09	–0.08	–0.056	0.04	–0.1
CTE	rs787080565	A997T	–0.83	–0.69	–1.53	–2.05	0.0
	rs791792095	P1022L	–0.75	–0.62	–0.629	–0.58	0.0
	rs335037856	R1095Q	–0.56	0.04	–0.115	0.15	–0.0
	rs336411058	E1114K	–0.57	–0.03	–0.499	–0.86	0.0
	rs320614499	T1123A	–0.76	0.15	–0.216	1.53	–0.0

Stabilizing mutations (ΔΔG > 0) are marked in blue, destabilizing mutations (ΔΔG < 0) in red, neutral (ΔΔG = 0) in gray. For PoPMuSIC, all ΔΔG values ​​are written with the opposite sign to match them with other services

Bioinformatic tools based on different algorithms were used to increase the accuracy of predicting the effect of substitutions on the energy stability of the tertiary structure of TERT. For some of the studied SNPs, a consistent score was obtained across all tools. Thus, it is worth paying attention to rs789641834 (L158M), rs325294961 (R354G), and rs324158660 (I516T), for which the corresponding amino acid substitutions are characterized by consensus negative ΔΔG values, which demonstrates their destabilizing effect on TERT (Table 5). In addition, the G227E, T270A, A997T, P1022L, and E1114K substitutions can be destabilizing. Amino acid substitutions R281W and R606L, which correspond to rs330770291 and rs698738374, stabilize the enzyme due to an increase in folding free energy, and they can probably be considered useful SNPs.

## Discussion

This study compared the degrees of identity and similarity of the pig *TERT* AAS and CDS with other phylogenetic species. As expected, the CDDs and AASs of the phylogenetically close to pig *Cetartiodactyla* species (cattle, goat, sheep) had the highest degrees of identity and similarity with corresponding pig *TERT* sequences. At the same time, rather high degrees of identity and similarity were also found for the pig *TERT* CDS and AAS with human *TERT*, which is important because the structural and functional features of the human *TERT* gene and the protein encoded by it are well-studied and can be used in the analysis of pig *TERT*. It is notable that the length of human *TERT* AAS is 1132 amino acid residues, which is very close to pig *TERT*, but a similar AAS length compared to human is due to a number of indels in the *TERT* gene and does not indicate a greater similarity of pig *TERT* to human *TERT* than to *Cetartiodactyla* species (cattle, goat, sheep).

It is also worth noting that the reference sequence of pig *TERT* entered the NCBI [48] database (NCBI Gene ID: 492280) has some differences from this sequence in Ensembl. Thus, according to NCBI data, the reference pig TERT protein consists of 1131 amino acid residues and, compared to the reference sequence in Ensembl, has the amino acid substitution R354G (the relevant rs325294961 is also considered in this study), as well as a single amino acid insertion (P700_P701insA). This may be the reason for some differences regarding the length of the AAS of pig *TERT* and the localization of individual SNPs when using the NCBI sequence as a reference.

According to the Ensembl data, there are 4 missense SNPs that correspond to the TEN domain of pig TERT (Table 2), while a total of 6 polymorphisms were detected in this gene region. For comparison, 336 polymorphisms (from which 178 missense) of human *TERT* and 75 polymorphisms (from which 56 missense) of cattle *TERT* genes are known in the same region. The significant difference in the number of SNPs found in the *TERT* genes of pigs, humans, and cattle is attributed to the varying degree of knowledge of these species. It should be noted that the influence of synonymous variants can also take place, for example, on the conformation and function of the protein, affecting post-transcriptional processing, or on the level of its synthesis through the mechanisms of mRNA translation [49, 50]. Functional data confirm that the TEN domain is required for telomerase recruitment to telomeres [51]. In humans, mutations in the DAT (Dissociates Activity of Telomerase) region in the TEN domain render the enzyme unable to function in vivo, but telomerase retains some catalytic activity in vitro [52]. This fact indicates the possibility of the influence of missense SNPs localized in the part of the pig *TERT* gene that encodes the TEN domain of telomerase on the activity of the enzyme.

The gene region that corresponds to the TRBD domain in the pig TERT contains 12 polymorphisms, out of which 5 are missense SNPs. Due to the low level of research on pig *TERT*, these data are unlikely to reflect the real level of polymorphism of this area in ​​the pig *TERT* gene because 330 and 63 polymorphisms have been established accordingly for human and cattle *TERT* in a similar area.

As for the RTD domain, there are a total of 19 SNPs known, with 5 of them being missense SNPs. According to data obtained for human *TERT*, this domain contains 7 conserved motifs [4]. Mutations in this domain lead to a decrease in enzyme activity [53]. Moreover, for yeast *TERT*, it was shown that in addition to mutations that disrupt the function of the enzyme and assembly of the telomerase complex [54, 55], there are mutations that lead to an increase in the length of telomeres [56]. These data give reason to expect that some of the missense SNPs found in the region of the pig *TERT* gene encoding the RTD domain can significantly affect the structure of the enzyme and its activity.

The gene region that corresponds to the CTE domain in pig telomerase is characterized by 5 missense polymorphisms. The CTE is known to participate in telomerase recruitment, and mutations in this domain do not affect telomerase activity in vitro but do not allow the enzyme to maintain telomere length in vivo [57].

In this study, a predictive assessment of the expected effect of pig *TERT* gene missense SNPs on telomerase function and stability was performed, for which sequence-based and structure-based methods were used. The results of evaluation by sequence-based methods showed that of all the considered SNPs, missense ones rs325294961, rs705602819, rs789641834, and rs706045634 can have such an effect.

As for rs325294961, this polymorphism involves an amino acid substitution of arginine to glycine (R354G) in the TRBD domain of telomerase, which may affect the interaction of TERT with telomerase RNA. There is the substitution of the cytosine nucleotide with a guanine nucleotide (CGG/GGG) in the first position of the codon. This agrees with the notion that substitutions of the first nucleotide of the codon have the greatest effect on protein structure. As a result of such a substitution, a charged amino acid is often replaced by an amino acid with the opposite charge [58]. In this case, arginine belongs to the basic amino acids and, thanks to the guanidine group, exhibits strong alkalic properties. This amino acid is able to form multiple hydrogen bonds with phosphate groups of nucleic acids. Glycine is a neutral amino acid, which differs significantly from arginine in terms of its chemical properties [59]. Thus, it is obvious that the substitution of arginine with glycine (R354G) can change the structural characteristics of telomerase and, probably, the affinity of the TRBD domain to telomerase RNA, which, in turn, can affect the catalytic activity of the enzyme. This assumption is confirmed by the evaluation of the effect of the R354G substitution in the TRBD domain, obtained using the bioinformatic tools SIFT, PROVEAN, PolyPhen-2, and SNAP2. According to involved software resources, this mutation is defined as having a significant effect on the enzyme. It is also worth noting that rs200191524 in an adjacent position of human *TERT* leads to the substitution of arginine for glutamine (R358Q), characterized by association with the phenotype according to ClinVar [60] data (RCV001219035.5).

The rs705602819 polymorphism corresponds to the amino acid substitution R629W in the RTD domain. In the same way as for the R354G, the substitution of arginine to tryptophan is associated with a substitution of a nucleotide in the first position of the codon (CGG/TGG). Indeed, the specified amino acids differ significantly in their chemical properties. Arginine is an alkalic hydrophilic amino acid; tryptophan is an aromatic amino acid that exhibits hydrophobic properties [59]. Predictive evaluation of all used sequence-based tools indicates a significant effect of the R629W substitution on the function of telomerase. Given that the RTD domain is responsible for the enzymatic reverse transcriptase function of telomerase, it can be expected that rs705602819 has the prospect of being used as a genetic marker associated with the activity of the enzyme. This thesis is also supported by the fact that the analyzed SNP in the pig *TERT* gene corresponds to rs1194223999 in the human *TERT* gene, which causes an identical amino acid substitution R631W in the analogous position of the enzyme. In addition, the polymorphisms leading to the substitutions R629W in the pigs and R631W in the humans are in a conserved region with similar AASs between different species, suggesting their similar influence. The rs1194223999 of human *TERT* gene shows a phenotypic effect consistent with ClinVar data (RCV001172450.2, RCV002411664.1) and scientific publications [61, 62]. In turn, current study bioinformatically predicted the functional effect of rs705602819 in pig *TERT* gene.

As mentioned above, the predictive scores for rs789641834, rs706045634, and rs705219838 by several sequence-based tools also suggest their possible effect on the functional characteristics of the enzyme (Table 4). The first two of these SNPs are located in the gene region corresponding to the TEN domain of telomerase. The rs789641834 is caused by a substitution of the first nucleotide of the codon (CTG/ATG) and is the cause of the corresponding amino acid substitution L158M in the AAS of the enzyme. Leucine is a typical non-polar aliphatic α-amino acid, and methionine is also a non-polar aliphatic α-amino acid but has a bonded sulfur atom that exhibits hydrophobic properties [59]. When leucine is replaced by methionine, the hydrophobicity of the latter may affect the spatial structure of the protein. Another rs706045634 as assessed by the SIFT, PolyPhen-2, and SNAP2 tools also demonstrates the possibility of influencing the functional properties of telomerase, leading to the amino acid substitution R201P in the same TEN domain. As for the last of the mentioned substitutions with possible effect, the rs705219838 is in the Linker region of the TERT enzyme and leads to the substitution T270I.

Comparison of the results of the predicted impact of the missense SNPs obtained by structure-based methods with the results obtained by sequence-based methods reveals certain regularities in the estimates. Thus, results of the sequence-based methods for rs789641834 (L158M) and rs325294961 (R354G) indicate that there is a potential “effect” of these polymorphisms on TERT functions (Table 4). At the same time, these polymorphisms predictably destabilize the structure of the TERT protein (Table 5). This allows us to make a general tentative conclusion about their significant conditionally deleterious effect on telomerase activity.

According to the results obtained using sequence-based methods, amino acid substitution R201P (rs706045634) refers to those that have a certain effect on the functional properties of telomerase. And according to structure-based methods, it is likely to contribute to increasing the stability of the enzyme. It can be assumed that this substitution affects the functional properties of the enzyme by increasing the stability of the tertiary structure of TERT.

Based on sequence-based methods, the substitutions R281W (rs330770291) and R606L (rs698738374) are estimated as neutral. This prediction is consistent with their assessment by structure-based methods, according to which they are likely stabilizing with respect to the tertiary structure of TERT. An increase in the stability of the TERT structure may contribute to a change in the activity of the enzyme.

Thus, changes in telomerase activity associated with the influence of rs789641834, rs325294961, and rs706045634 (based on both sequence and structure prediction) and rs705602819 (based on strong sequence prediction) can result in changes in processes associated with cell proliferation, which, as can be assumed, affect physical parameters.

Given the important biological role of telomerase and the relationship of its activity, as mentioned above, with the manifestation of traits of livestock animals, the *TERT* gene is considered in this study as a candidate gene that exhibits biological functional impact. The results of bioinformatic evaluation help to conduct a target selection for associative analysis studies of those SNPs for which the effect on the structure and function of the protein is demonstrated in silico. In this case, out of 23 missense polymorphisms analyzed in the pig *TERT* gene, 4 SNPs (rs789641834, rs325294961, rs706045634, and rs705602819) are the most promising candidate genetic markers.

Noted SNPs should be tested in associative analysis studies to establish their actual effect on animal performance. If the results of such studies demonstrate their significant association with such traits, they can obviously be used in MAS and can be considered direct genetic markers. The mutations corresponding to them can claim to be classified as causative, since they directly affect the functional and structural characteristics of TERT, which, in turn, changes the activity of the enzyme. Those SNPs for which the corresponding amino acid substitutions, according to the results of bioinformatic analysis, did not reveal an effect on the characteristics of TERT, but in the associative analysis performed for them show an effect on productive traits, obviously, can be considered linkage disequilibrium genetic markers.

This approach seems to be useful for searching genetic markers of candidate genes. The *TERT* gene in this study served as a model object for its demonstration.

## Conclusions

Pig productivity depends on health, productive lifespan, resistance to stress, and environmental factors. The use in breeding programs of genetic markers associated with these key qualities of farm animals will improve the selection and economic aspect of pig breeding. Such markers can be developed based on the polymorphism of the telomerase gene, which is involved in the control of important biological processes.

The organization of the pig *TERT* gene was analyzed in comparison with *TERT* in other biological species using bioinformatic methods. Significant similarities between CDS and AAS of the pig *TERT* with those sequences of other species have been established. The comparison was also made with human *TERT* in the context of the structural-functional domains of the enzyme and the distribution of SNPs. Due to the high similarity between pig and human TERT, a three-dimensional structure of pig TERT was obtained using the homology modeling method.

According to the results of bioinformatic analysis of 23 missense SNPs of the pig telomerase gene, a predictive effect of rs789641834, rs706045634, rs325294961 and rs705602819 on the structural and functional parameters of the enzyme were established. The *TERT* gene, given the important role of telomerase enzyme, which it encodes, can be considered a candidate gene involved in the control of biological and productive traits. The bioinformatic assessment of these SNPs enables a targeted selection of polymorphisms that may serve as potential genetic markers, thus the possibility of their application in MAS should be further investigated through associative analysis studies.

## Methods

### Analysis of the primary structure of the *TERT* gene and protein

The coding sequence (CDS) and the amino acid sequence (AAS) corresponding to the pig *TERT* gene (Ensembl ID: ENSSSCG00000017118, duroc breed) obtained from Ensembl [5] were used as references. Canonical CDSs and AASs of the *TERT* of the following organisms were used for comparative analysis and alignments: human (Ensembl ID: ENSG00000164362); model organisms: mouse (Ensembl ID: ENSMUSG00000021611), rat (Ensembl ID: ENSRNOG00000025327), African clawed frog (NCBI Gene ID: 373635), zebrafish (Ensembl ID: ENSDARG00000042637), takifugu (Ensembl ID: ENSTRUG00000014198); agricultural species: cattle (Ensembl ID: ENSBTAG00000012567), sheep (Ensembl ID: ENSOARG00020010728), goat (Ensembl ID: ENSCHIG00000008229), horse (NCBI Gene ID: 100630695, isoform X1), donkey (NCBI Gene ID: 106835880).

Pairwise alignments of CDSs and AASs were performed using the Needleman-Wunsch algorithm [63] in the EMBOSS Needle [64]. According to the alignment results, the degrees of identity between pigs and other biological species CDSs were determined, as well as the degrees of identity and the degree of similarity between the pigs and AASs of other biological species (the degree of identity in this study refers to the percentage of identical nucleotides or amino acids in CDSs and AASs respectively; the degree of similarity refers to the percentage of amino acids in AASs with alignment score > 0 accordingly to EBLOSUM62 substitution matrix).

SNPs in the pig *TERT* gene, along with their corresponding rsIDs, were identified using data from the Ensembl database. To evaluate the distribution of polymorphisms across pig TERT domains, multiple alignment of pig *TERT* AAS was conducted with corresponding sequences of cattle (a phylogenetically related species) and human (whose TERT domain structure is known, sourced from UniProt ID: O14746) sequences. Alignment was performed using MUSCLE algorithm [65] in MEGA11 software [66].

For pig breeds with available whole genome sequencing data, a phylogenetic analysis was conducted using the relevant CDSs. The following breeds were included in the analysis: bamei (Ensembl ID: ENSSSCG00050053174), berkshire (Ensembl ID: ENSSSCG00065076605), duroc (reference pig *TERT* sequence), hampshire (Ensembl ID: ENSSSCG00035007219), jinhua (Ensembl ID: ENSSSCG00060012419), landrace (Ensembl ID: ENSSSCG00045034770), largewhite (Ensembl ID: ENSSSCG00025035362), meishan (Ensembl ID: ENSSSCG00040053333), pietrain (Ensembl ID: ENSSSCG00055017091), rongchang (Ensembl ID: ENSSSCG00030034123), tibetan (Ensembl ID: ENSSSCG00015056815), USMARC (Ensembl ID: ENSSSCG00070009936). The MUSCLE algorithm in MEGA11 software was used to perform a multiple alignment of the *TERT* CDSs of these breeds. Subsequently, a phylogenetic tree was constructed using the Maximum Likelihood method and the JTT matrix-based model [67]. Missense polymorphisms in pig *TERT* gene were included in the further analysis because they can lead to amino acid substitutions in AAS and potentially impact the function of the protein encoded by the *TERT* gene. For all missense polymorphisms, allelic variants corresponding to each of the studied 12 pig breeds were identified.

### Assessment of the SNPs using sequence-based methods

This approach involved evaluating the impact of missense substitutions on TERT protein functions using computational tools that utilize *TERT* AAS as input data. Sequence homology-based tools such as SIFT [37] and PROVEAN [38, 39] determine the impact of missense substitutions based on the alignment of the input sequence with homologous sequences and assessment of position conservatism, where there are suitable substitutions. A set of homologous sequences that were selected using BLAST [68] in the non-redundant UniRef90 database [69] were used as related sequences in SIFT evaluation. PANTHER [40] is a predictive tool that evaluates position-specific evolutionary preservation as the length of time (in millions of years) a position in protein has been preserved, i.e. determines the conservatism of the position in which the substitutions take place. PolyPhen-2 [41, 42] and SNAP2 [43, 44] are the tools that utilize various characteristics of substitution to assess its functional impact.

### Assessment of the SNPs using structure-based methods

This approach involved evaluating the impact of polymorphisms on the stability of the TERT protein based on the data of the three-dimensional structure of the protein. Since no such experimental structure has been established for the pig TERT, homology modeling was used. Homology modeling of the pig TERT was performed using MODELLER 10.3 software [70]. The cryo-EM map of the human telomerase-DNA-TPP1 complex (PDB ID: 7QXA [71] was used as a template structure for modeling. The obtained as a result of homology modeling three-dimensional pig structure was minimized in the Amber ff14SB force field [72] according to the built-in protocol in the UCSF Chimera software [73]. Evaluation of the minimized model was carried out using the ProSA-web service [74, 75]. The three-dimensional structure was visualized in free PyMOL software [76]. To assess the influence of SNPs on the energy stability of the protein, bioinformatic tools with different predictive algorithms were used: I-Mutant 3.0 is the support vector machine (SVM)-based tool [77]; PoPMuSiC is the server that makes predictions using a linear combination of statistical potentials [78–80]: mCSM is the tool based on graph-based structural signatures [81]; SDM2 is the server that uses conformationally-constrained environment-specific substitution tables for prediction [82]; DDGun is the tool that makes prediction through a linear combination of scores derived from sequence and structural evolutionary features [83]. Pig TERT protein structure minimized in UCSF Chimera, as mentioned above, was used as input in all services except SDM2; for making a prediction using the SDM2 service, a structure with minimization in the GROMOS96 43B1 force field [84] performed in the Swiss-PdbViewer 4.1.0 [85] was used to reduce the size of the input file following requirements of the SDM2 server.

## Data Availability

Models of the structure of *Sus scrofa* (pig) telomerase reverse transcriptase (TERT) are available in ModelArchive (modelarchive.org) with the accession codes ma-ydbtw, ma-p89hn. Other datasets used and/or analysed during the current study are available from the corresponding author on reasonable request.

## Abbreviations

aa: Amino acids
AAS: Amino acid sequence
CDS: Coding sequence
CTE: C-terminal domain
PM: Polymorphism
MAS: Marker-associated selection
RTD: Reverse transcriptase domain
SNP: Single-nucleotide polymorphism
TEN: N-terminal domain
TER: Telomerase RNA component
TERT: Telomerase reverse transcriptase
TRBD: Telomerase RNA-binding domain
